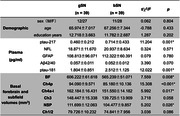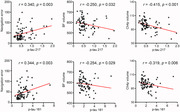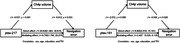# Ch4p volumes mediate the relationship between plasma p‐tau and spatial navigation

**DOI:** 10.1002/alz.085803

**Published:** 2025-01-09

**Authors:** Qian Chen

**Affiliations:** ^1^ Nanjing Drum Tower Hospital, Nanjing, Jiangsu China

## Abstract

**Background:**

To investigate the relationships among plasma biomarkers, basal forebrain, and spatial navigation.

**Method:**

A total of 78 participants were enrolled, including 23 normal controls (NC), 38 subjective cognitive decline (SCD), and 17 mild cognitive impairment (MCI) patients. According to the spatial navigation distance errors in the human version of the Morris Water Maze, the whole cohort was divided into the good spatial navigation performance (gSN) group and the bad spatial navigation performance (bSN) group, with 39 cases in each group. The plasma biomarkers of p‐tau217, neurofilament light chain (NfL), glial fibrillary acidic protein (GFAP), β‐amyloid (Aβ) 42/40, and p‐tau181 were measured by single molecule array (SimoA). Basal forebrain (BF) volumes were calculated, as well as the subfields of Ch4p, Ch4a‐i, Ch3, NSP, and Ch1/2. Correlation and mediation analyses were performed to assess the relationships among plasma biomarkers, basal forebrain, and spatial navigation.

**Result:**

The gSN and bSN groups did not differ significantly in sex, age, and education years. Compared to the gSN group, the bSN group showed increased p‐tau217 and p‐tau181. The differences in NfL, GFAP, and Aβ42/40 levels were not significant. The bSN group showed significant volume reduction in the basal forebrain, especially the Ch4p subfield. The plasma p‐tau217 and p‐tau181 levels were positively correlated with navigation errors (r = 0.340, p = 0.003; r = 0.344, p = 0.003; respectively), while negatively correlated with basal forebrain (r = ‐0.250, p = 0.032; r = ‐0.254, p = 0.029; respectively) and Ch4p volumes (r = ‐0.415, p <0.001; r = ‐0.319, p = 0.006; respectively). Mediation analysis showed that the Ch4p volumes mediated the relationships between plasma p‐tau217/181 and navigation errors.

**Conclusion:**

These findings suggest that spatial navigation may serve as sensitive markers for the early identification of Alzheimer’s disease p‐tau pathology. The results may also indicate the relationships among “plasma p‐tau → basal forebrain atrophy → spatial navigation dysfunction”.